# Lipid Nanoparticle Delivery of mRNA and siRNA for Concurrent Restoration of Tumor Suppressor and Inhibition of Tumorigenic Driver in Prostate Cancer

**DOI:** 10.1021/acsnanoscienceau.4c00066

**Published:** 2024-12-26

**Authors:** Ryan A. Farokhzad, Jing Luo, Li Jia, Yang Zhang, Jinjun Shi

**Affiliations:** † Center for Nanomedicine and Department of Anesthesiology, Perioperative and Pain Medicine, 1811 Brigham and Women’s Hospital, Harvard Medical School, Boston, Massachusetts 02115, United States; ‡ Department of Urology, 1811 Brigham and Women’s Hospital, Harvard Medical School, Boston, Massachusetts 02115, United States

**Keywords:** mRNA, siRNA, lipid nanoparticle, prostate cancer, combination therapy

## Abstract

Cancer is commonly caused by a gain of function in proto-oncogenes and a simultaneous loss of function in tumor suppressor genes. Advanced prostate cancer (PCa) is often linked with changes in the activity or expression of phosphatase and tensin homologue deleted on chromosome 10 (PTEN), a well-known tumor suppressor, and androgen receptor (AR), a pro-tumorigenic transcription factor. However, no therapies exist for the simultaneous correction of tumorigenic promotion and suppressor depletion. Here, we report that concurrent PTEN restoration and AR silencing by lipid nanoparticle (LNP) delivery of PTEN messenger RNA (mPTEN) and AR small interfering RNA (siAR) elicited synergistic therapeutic effects in PCa cells. We screened various LNP formulations for the optimal delivery of both RNAs. In C4-2 and LNCaP cells, both of which are AR-positive and PTEN-null PCa cell lines, the combinatorial treatment of siAR and mPTEN LNPs resulted in much stronger cytotoxicity in vitro than the treatment of either alone. Western blot analyses revealed concurrent regulation of phosphatidylinositol 3-kinase-protein kinase B (PI3K-AKT) and extracellular signal-regulated kinase (ERK) pathways, leading to increased caspase-3 cleavage-mediated apoptosis. Our findings suggest that the strategy of RNA-mediated concurrent restoration of tumor suppressors and inhibition of tumorigenic drivers could lead to the more effective treatment of PCa and potentially other malignancies.

## Graphical abstract

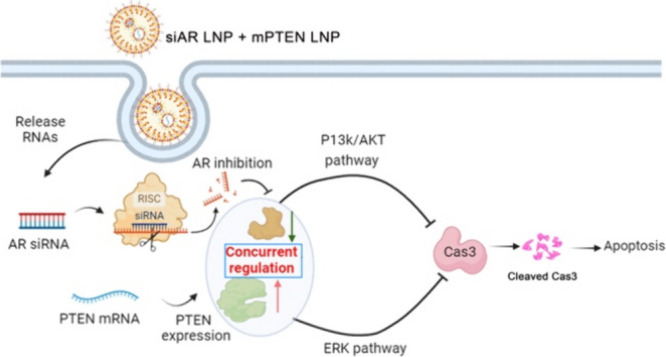

## 1 Introduction

The spectrum between health and disease is often associated with an imbalance between cellular signals that promote activation or inactivation of distinct biological pathways.^1^ In cancers, this imbalance results from the dysregulation of pro-tumorigenic and tumor-suppressing pathways.^2–4^ For example, the progression of prostate cancer (PCa), the second leading cause of cancer death in men, is often associated with the loss of tumor suppressors, such as phosphatase and tensin homologue deleted on chromosome 10 (PTEN), as well as the activation of tumorigenic drivers such as androgen receptor (AR).^5–8^ Through negative regulation of the phosphatidylinositol 3-kinase-protein kinase B (PI3K-AKT) pathway, PTEN inhibits tumor proliferation, metastasis, and angiogenesis and also induces apoptosis.^9,10^ Despite PCa’s heterogeneous genetic profile, loss of functional PTEN is found in roughly 20% of early-stage PCa and approximately 50% of castration-resistant PCa, underscoring its prevalence in the disease.^11,12^ On the other hand, the AR gene is the most frequently altered gene in castration-resistant PCa, with an alteration rate of approximately 63% in a multi-institutional cohort of 150 individuals.^13^ The most common alteration is AR amplification, and AR is one of the most therapeutically targeted oncogenic drivers in PCa. Current therapies for advanced PCa mainly focus on AR signaling inhibition, which, however, is often limited by the development of therapeutic resistance.^14,15^ In addition, AR inhibition does not address the underlying tumor suppressor loss.^16–18^ Similarly, direct targeting of the PI3K-AKT pathway may be inadequate for restoring the regulatory feedback provided by functional PTEN.^7,19,20^ These limitations highlight the need for an integrative approach to re-establish the balance between cellular anti- and pro-growth signals mediated by PTEN and AR, respectively, which could lead to the development of a novel advanced PCa therapy with more potent efficacy.

Advances in RNA technologies have recently resulted in multiple medicines for clinical use, including small interfering RNA (siRNA) for silencing protein expression and messenger RNA (mRNA) for inducing protein expression. Six siRNA therapeutics and three mRNA vaccines have been approved by the Food and Drug Administration (FDA), and numerous potential RNA therapeutics for cancer and other diseases are under preclinical and clinical development.^21–24^ The use of siRNA to silence the expression of amplified or mutated tumorigenic proteins has been extensively reported.^25^ Additionally, we and others have recently applied mRNA delivery to restore the tumor suppressor function. We demonstrate the therapeutic potential of tumor suppressor (e.g., PTEN and p53)-coded mRNA in inhibiting tumor growth, enhancing antitumor immunity, and improving other therapies such as immune checkpoint blockade.^26–32^ We hypothesize that using mRNA and siRNA to concurrently restore tumor suppressors (e.g., PTEN) and inhibit tumorigenic drivers (e.g., AR) could represent a unique strategy for correcting both aspects of the cellular signaling imbalance for the highly effective treatment of PCa.

In this study, we developed and screened distinct lipid nanoparticle (LNP) formulations for the optimal delivery of siRNA and mRNA, ensuring minimal cytotoxicity and efficient cellular uptake and RNA transfection. We investigated combined delivery, defined as the coadministration of two distinct LNPs, carrying either siAR or mPTEN, as well as codelivery, defined as the administration of a single LNP, carrying both siAR and mPTEN. We evaluated the efficacy of this dual-targeting approach in AR-positive and PTEN-null PCa cells in vitro by examining cell viability, apoptosis, and key signaling pathways related to PTEN and AR. Cytotoxicity results revealed that the combined delivery of PTEN mRNA (mPTEN) and AR siRNA (siAR) LNPs exhibited a combination index (CI), a quantitative measure of the combined effects of two drugs, below 0.5 with select RNA concentrations, indicating a strong synergistic effect. Flow cytometry analysis also showed a significant increase in early and late apoptosis with over 60% of cells in the combination group undergoing apoptosis. Mechanistic studies demonstrated that the concurrent restoration of PTEN and inhibition of AR induce significant apoptosis through caspase-3 activation, decrease cell proliferation by inhibiting the PI3K-AKT and extracellular signal-regulated kinase (ERK) signaling pathways, and disrupt AR-mediated pro-growth signaling. We expect that this approach of LNP delivery of siRNA and mRNA could be robustly expanded to many other tumor suppressors and tumorigenic drivers for a fundamental biological understanding and therapeutic development.

## 2 Materials and Methods

### 2.1 Materials

D-Lin-MC3-DMA (MC3, MCE, CAS No. 1224606-06-7), SM-102 (MCE, CAS No. 2089251-47-6), 1,2-dioleoyl-*sn-glycero*-3-phosphoethanolamine (DOPE, Cayman Chemical, CAS No. 4004-05-1), cholesterol (Sigma, CAS No. 57-88-5), and 1,2-dimyristoyl-*rac-glycero*-3-methoxypolyethylene glycol-2000 (DMG-PEG2000, Avanti, CAS No. 880151P) were purchased for LNP formulation. Small interfering RNAs targeting luciferase (siLuc) and androgen receptor (siAR) were synthesized by Horizon Discovery. Messenger RNAs targeting GFP (mGFP) and PTEN (mPTEN) were synthesized by TriLink Bio Technologies. The AlamarBlue assay (Thermo Fisher, Cat. No. A50100) was used for cell viability assays, and the Annexin V-FITC-PI kit (Thermo Fisher, Cat. No. A13199) was employed for apoptosis detection. Antibodies used for Western blotting included anti-AR (CST, Cat No. 5153), anti-PTEN (Biolegend, Cat No. 655002), anti-pERK (CST, Cat No. 9101), anti-pAKT (CST, Cat No. 4060), anticleaved caspase-3 (CST, Cat No. 9661), and antiactin (CST, Cat No. 4967).

### 2.2 Preparation of RNA LNPs

LNPs were formulated by the pipetting technique to encapsulate siRNA or mRNA. MC3/SM-102, DOPE, cholesterol, and DMG-PEG2000 in different ratios were dissolved in ethanol. For the optimization of the nitrogen-to-phosphorus (N:P) ratio for siRNA and mRNA delivery, we varied the amount of ionizable lipids while keeping the RNA amount constant to achieve ratios of 4, 5, and 6. The lipid mixture was rapidly mixed by pipetting with an aqueous solution of siRNA or mRNA in a sodium acetate buffer (pH 4.0) at a ratio of 3:1. The resultant LNPs were immediately diluted in PBS and dialyzed against PBS (pH 7.4) to remove ethanol and adjust the pH.

For LNP screening, 12 formulations were prepared for each siRNA and mRNA payload by varying the LNP components (Table S1). The ionizable lipids, MC3 and SM-102, were used at different molar percentages along with DOPE, cholesterol, and DMG-PEG2000. Each formulation was prepared as described above.

### 2.3 Cell Culture and Transfection

C4-2 and LNCaP PCa cells were cultured in RPMI-1640 medium supplemented with 10% fetal bovine serum (FBS) and 1% penicillin-streptomycin. The cells were maintained in a humidified atmosphere at 37 °C with 5% CO_2_. For transfection experiments, cells were seeded into 96-well culture plates to achieve a confluence of ∼60–70%. LNPs containing siRNA or mRNA were incubated with the cells for 24 h. Subsequently, the medium was replaced, and the cells were cultured for another 24 h before measuring RNA transfection efficiency. For the siLuc silencing evaluation, cells were lysed using a passive lysis buffer. Luciferase activity was measured by adding a luciferase substrate to the lysate and measuring luminescence with a luminometer. For mGFP expression detection, cells were visualized by using a fluorescence microscope. Images were captured from multiple fields to ensure representative sampling, and the fluorescence intensity was quantified using image analysis software by measuring the mean fluorescence intensity (MFI) of GFP-positive cells.

### 2.4 Combination Treatments and Synergy Evaluation of siAR and mPTEN LNPs

Cells were treated with varying concentrations of siAR LNPs (5, 10, 20, and 40 nM siRNA) and mPTEN LNPs (62.5, 125, 250, and 500 ng/mL mRNA) individually to establish the dose–response curves. For combination treatments, cells were cotreated with siRNA and mRNA LNPs at all possible concentration combinations. LNPs were incubated with the cells for 24 h, followed by medium replacement and another 24 h incubation. Cell viability was measured using the AlamarBlue assay. The synergistic effects of concurrent delivery were evaluated using the Chou–Talalay method.^33^ Cell viability data from combination treatments were analyzed by using CompuSyn software to calculate the CI. We adhered to the standard approach for combination analysis, as recommended by Chou–Talalay, and no modifications were made to the underlying principles or calculations in the software. The CI shows the extent to which a given interaction of drugs will produce a synergistic, addictive, or antagonistic result. A CI value less than 1 indicates synergy; a value equal to 1 indicates an additive effect; and a value greater than 1 indicates antagonism.

### 2.5 Apoptosis Effect Evaluation

Live-cell imaging was performed by using Calcein-AM staining. After treatment with RNA LNPs, cells were washed with PBS and incubated with 2 μM Calcein-AM in PBS for 30 min at 37 °C. Fluorescence images were captured using a fluorescence microscope. The MFI of Calcein-AM was quantified to assess the cell viability.

Apoptosis was also evaluated using an Annexin V-FITC/PI apoptosis detection kit. Treated cells were harvested, washed twice with cold PBS, and resuspended in binding buffer at a concentration of 1 × 10^6^ cells/mL. 5 μL of Annexin V-FITC and 5 μL of PI were added to 100 μL of the cell suspension and then incubated for 15 min at room temperature in the dark. Samples were then diluted with 400 μL of binding buffer and analyzed by flow cytometry within 1 h. Data was collected for at least 10,000 events per sample, and the percentages of viable, early apoptotic, late apoptotic, and necrotic cells were determined using appropriate quadrants in the flow cytometry plots.

### 2.6 Western Blot Analysis

Cells were lysed in a radioimmunoprecipitation assay buffer containing protease and phosphatase inhibitors. Protein concentrations were determined using the BCA protein assay kit. Equal amounts of protein (20–30 μg) were separated by SDS-PAGE on 10% gels and transferred onto polyvinylidene fluoride membranes. Membranes were blocked with 5% nonfat milk in Tris-buffered saline with 0.1% Tween-20 for 1 h at room temperature. They were then incubated overnight at 4 °C with primary antibodies against AR, PTEN, pAKT, pERK, cleaved caspase-3, and β-actin. After washing with TBST, membranes were incubated with HRP-conjugated secondary antibodies for 1 h at room temperature. Bands were visualized using an enhanced chemiluminescence detection system and quantified using densitometry software.

### 2.7 Statistical Analysis

Statistical significance was determined using one-way analysis of variance (ANOVA) followed by Tukey’s post hoc test for multiple comparisons. A *p* value less than 0.05 was considered statistically significant. GraphPad Prism software was used for all statistical analyses and graphing.

## 3 Results and Discussion

### 3.1 Optimization of LNP Formulation for Delivery of siRNA and mRNA

The optimization of the LNP formulation was first conducted by varying the nitrogen-to-phosphorus (N:P) ratio for both siRNA and mRNA delivery. Three N:P ratios (4, 5, and 6) were tested by increasing the amount of ionizable lipid (MC3) while maintaining a constant RNA amount. An optimal N:P ratio is critical for achieving efficient encapsulation, protecting RNA, and effectively and safely delivering RNA-encapsulated LNPs to target cells. The model luciferase siRNA (siLuc) was chosen for the formulation of siLuc LNPs (Figure 1A). The average size of the siLuc LNPs was approximately 100 nm, and no significant differences were observed between N:P ratios of 4, 5, and 6 (Figure 1B). Cell viability analysis showed that all siLuc LNP formulations had minimal cytotoxicity at a concentration of 40 nM. Among the different N:P ratios, the ratio of 4 resulted in the lowest cytotoxicity compared to higher ones, yet the differences were not statistically significant (Figure 1C). Luciferase expression slightly decreased as the N:P ratio increased, though siRNA silencing was efficient at all the tested N:P ratios (Figure 1D). For mRNA delivery, the model green fluorescent protein mRNA (mGFP) was chosen for testing the LNPs (Figure 1E). The average size of mGFP LNPs was approximately 110 nm, and no significant differences were noted among the tested N:P ratios (Figure 1F). Cell viability results demonstrated minimal cytotoxicity across all mGFP LNP formulations (Figure 1G); however, an N:P ratio of 4 resulted in the lowest cytotoxicity. Additionally, an N:P ratio of 4 showed roughly 2-fold higher GFP expression compared to ratios of 5 and 6, suggesting better mRNA delivery (Figure 1H). This high GFP expression at an N:P ratio of 4 with similar size, cell viability, and luciferase expression distinguished an N:P ratio of 4 from a ratio of 5 or 6. Our data shows that an N:P ratio of 4 was the optimal condition, which provides the best balance for both efficient siRNA/mRNA delivery and minimal cytotoxicity.

**Figure 1:**
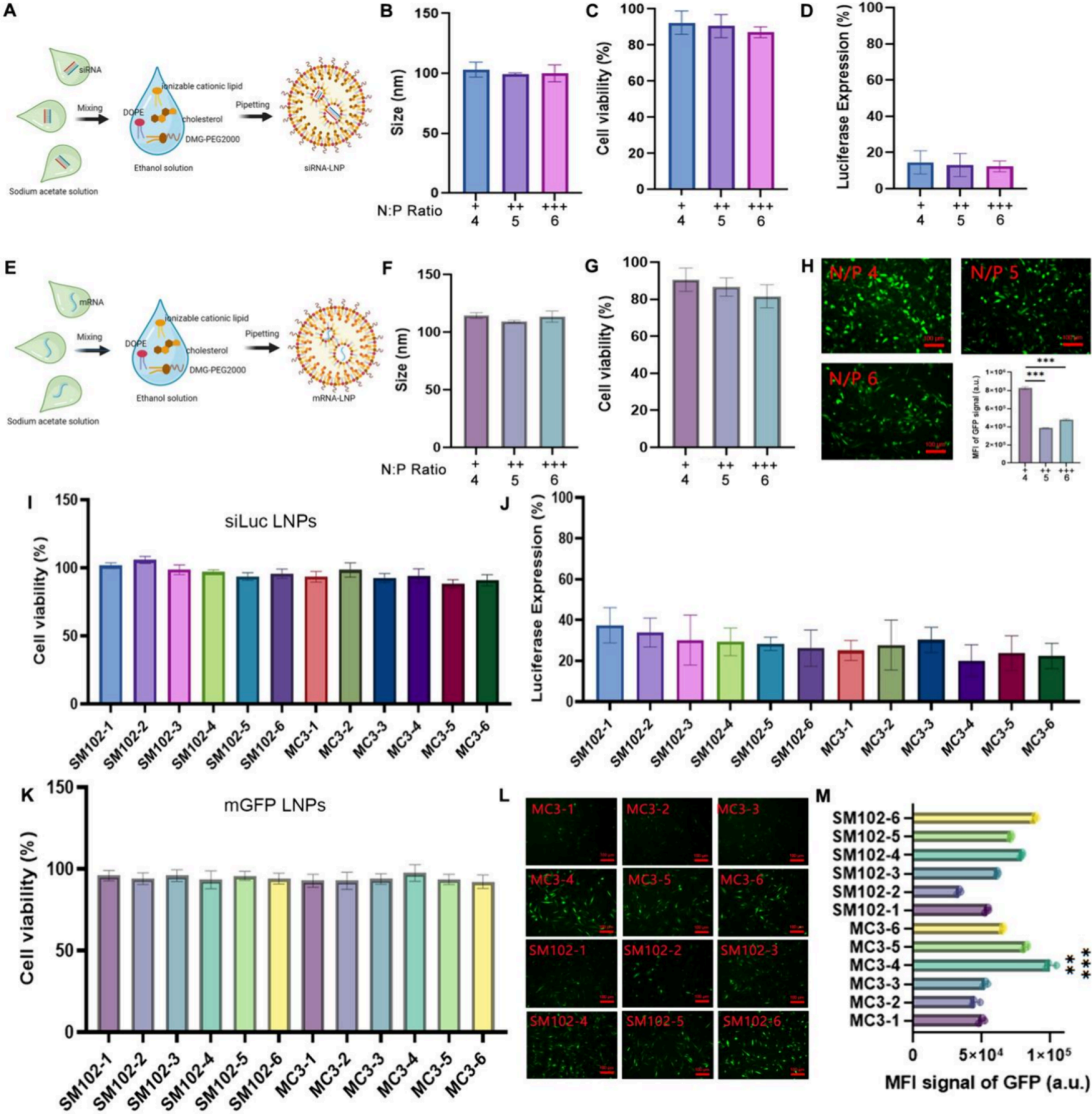
Characterization and screening of LNP formulations for siRNA and mRNA delivery. (A) Schematic representation of the formulation process for siRNA LNPs using different combinations of ionizable lipids, cholesterols, and other components. (B) Size characterization of siRNA LNPs measured by dynamic light scattering (DLS). (C) Viability of C4-2 PCa cells after treatment with siRNA LNPs at 40 nM concentration. (D) Evaluation of luciferase silencing efficiency in C4-2 cells treated with different siLuc LNP formulations. (E) Schematic representation of the formulation process for mRNA LNPs, detailing the mixing of ionizable lipid, cholesterol, and other components with mRNA. (F) Size characterization of mRNA LNPs by DLS. (G) Cell viability following treatment with mRNA LNPs at 500 ng/mL. (H) Representative fluorescence images of cells treated with mGFP LNPs. Scale bar: 100 μm. (I) Cell viability of different siRNA LNP formulations. (J) Luciferase expression after treatment with different siLuc LNP formulations. (K) Cell viability of mRNA LNP formulations. (L) Representative images of GFP expression in cells treated with different mRNA LNP formulations. Scale bar: 100 μm. (M) MFI of GFP signal in cells from (L). **, *p* < 0.01; ***, *p* < 0.001 (*n* = 4). ** in (M) means statistical significance of MC3-4 vs SM102-1, SM102-3, SM102-4, SM102-5, SM102-6, MC3-5, and MC3-6; *** means statistical significance of MC3-4 vs SM102-2, MC3-1, MC3-2, and MC3-3.

Following N:P ratio screening, a library of 12 LNP formulations was prepared for each siRNA and mRNA delivery by varying the composition of two distinct ionizable lipids, DOPE, cholesterol, and DMG-PEG2000 to identify the optimal LNP formulation for siRNA and mRNA delivery (Tables S1 and S2). The two ionizable lipids examined were MC3 and SM-102, both of which have been clinically validated for RNA delivery. MC3 was used in the LNP delivery system of the first FDA-approved siRNA drug, Onpattro, while SM-102 was used as an ionizable lipid in the mRNA LNP-based COVID-19 vaccine. For siRNA delivery, the size of these siLuc LNP formulations ranged from ∼79 to ∼151 nm. As exhibited in Figure 1I, all siLuc LNP formulations demonstrated a high cell viability. Luciferase silencing efficiency ranged from ∼63 to ∼80%, with MC3-4 showing the best silencing (Figure 1J). Furthermore, siRNA encapsulation efficiency (EE) ranged from ∼56 to ∼76%, with MC3-4 having an EE of ∼73% (Table S1). These results indicate that all tested LNP formulations could effectively deliver siRNA while maintaining a low cytotoxicity. Although the results show minimal differences in luciferase silencing, this may reflect the robustness of these formulations in delivering siRNA, while their efficiency in mRNA delivery varies more significantly. For mRNA delivery, the size of all mGFP LNP formulations ranged from ∼89 to ∼144 nm, and similar results were obtained with high cell viability for these mGFP LNP formulations (Figure 1K). GFP expression analysis by fluorescence microscopy revealed that MC3-4 and SM-102-6 achieved the highest levels of GFP expression (Figure 1L,M). The mRNA EE for all formulations ranged from ∼86 to ∼94%, with MC3-4 having an EE of 94% (Table S2). Based on the combined analyses of cell viability, luciferase silencing, GFP expression, and EE, the MC3-4 formulation emerged as the optimal candidate for delivering both siRNA and mRNA, which consisted of 13.5% DOPE, 50% MC3, 35% cholesterol, and 1.5% DMG-PEG2000. The screening of different LNP formulations demonstrated that the lipid composition plays a crucial role in determining the efficacy of RNA delivery.

### 3.2 Evaluation of Concurrent Treatment of siAR and mPTEN LNPs

The concurrent treatment of siAR and mPTEN LNPs in AR-positive and PTEN-null PCa cell lines, C4-2 and LNCaP, was evaluated to determine the combined efficacy of AR inhibition and PTEN restoration. We first tested the cytotoxicity for the combined delivery of control siLuc LNPs and mGFP LNPs. The results demonstrated that even at high concentrations (e.g., 40 nM for siLuc and 500 ng/mL for mGFP), cell viability remained above 80% in both C4-2 and LNCaP cell lines (Figure S1). This indicates that the selected LNP formulations are biocompatible for studying the in vitro antitumor effects of siAR and mPTEN LNPs. The siAR LNPs and mPTEN LNPs (with an average size of ∼104 and ∼120 nm, respectively, Figure S2) were then incubated with C4-2 and LNCaP cells for cell viability evaluation.

Individual dose–response experiments showed that siAR LNPs had minimal cytotoxicity, with cell viability ranging from 93 to 97% across different siAR concentrations (5–40 nM) for C4-2 cells (Figure 2A). In contrast, mPTEN LNPs exhibited concentration-dependent cytotoxicity, with low cytotoxicity at 62.5 and 125 ng/mL (5.05% ± 3.71% and 9.83% ± 0.75%, respectively) but increased cell death at 250 and 500 ng/mL (29.15% ± 5.75% and 53.40% ± 1.07%, respectively) (Figure 2B). When mPTEN LNPs were coadministrated with siAR LNPs, there was a dose-dependent enhancement of cytotoxicity compared to individual treatments (Figure 2C). For instance, at 250 ng/mL mPTEN, cotreatment with 5, 10, 20, and 40 nM siAR reduced cell viability to 69.3, 42.3, 23.1, and 18.3%, respectively, compared to 29.2% with mPTEN alone, indicating significantly enhanced efficacy. The CI values for different combinations of mPTEN and siAR were calculated, with values below 1 indicating synergy and values below 0.5 indicating strong synergy. The CI heatmap showed that mPTEN combined with siAR resulted in strong synergy, particularly at higher doses of both components. Notably, the best synergistic effect was observed with siAR at 20–40 nM and mPTEN at 250–500 ng/mL, with CI values of 0.23–0.39 (Figure 2D). This strong synergistic effect at higher concentrations may be attributed to the optimal balance between AR silencing and PTEN restoration. To further investigate the synergistic effects between siAR LNPs and mPTEN LNPs, the additive index (*f*_additive_) as well as the combination index (*f*_combination_) were calculated using the reported method.^34^ The *f*_additive_ = *f*_siAR_ × *f*_mPTEN_ (*f*_siAR_ is cell viability in the siAR LNP group, and *f*_mPTEN_ is cell viability in mPTEN LNP group), while the *f*_combination_ is cell viability in siAR and mPTEN LNPs combination therapy. Figure 2E shows the comparison between the calculated values of *f*_additive_ and that of *f*_combination_. It was observed that the *f*_additive_ values gradually exceeded the *f*_combination_ values with increased mPTEN concentrations, indicating a synergistic effect between siAR and mPTEN therapy. These results confirm the hypothesis that concurrently addressing the restoration of tumor suppressors and inhibition of tumorigenic drivers could lead to a highly potent antitumor effect. Furthermore, similar results were observed in LNCaP cells (Figure S3), where the combination of 20 nM siAR with 250 or 500 ng/mL mPTEN led to cell death rates of 70.11% ± 9.35% and 84.25% ± 8.95%, respectively, indicating consistency across both cell lines. While the cell viability measurements and CI values demonstrate that the 40 nM siAR and 500 ng/mL mPTEN had the highest degree of cell killing and synergy, for our subsequent studies, we chose to pursue siAR at 20 nM and mPTEN at 250 ng/mL to balance a high degree of synergistic efficacy while retaining a sufficient number of viable cells for Western blot and flow cytometry analyses.

**Figure 2:**
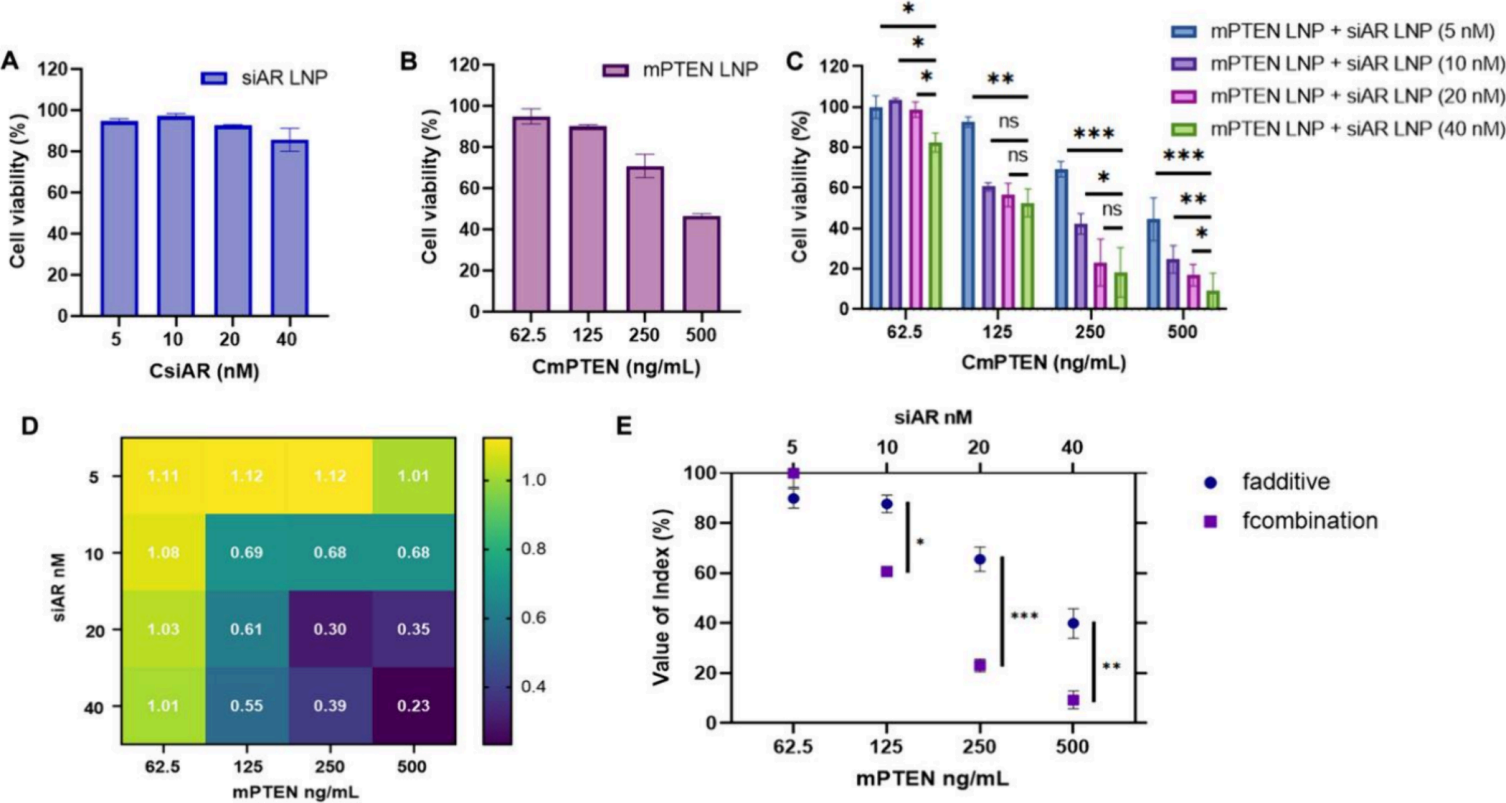
Evaluation of the synergistic effect by concurrent treatment of siAR and mPTEN LNPs in C4-2 cells. (A, B) Dose-responsive cell viability of PCa cells after treatment with (A) siAR LNPs and (B) mPTEN LNPs at varying concentrations. (C) Cell viability following cotreatment with siAR and mPTEN LNPs at different siAR concentrations (5, 10, 20, and 40 nM) combined with different mPTEN concentrations (62.5, 125, 250, and 500 ng/mL). (D) Heatmap showing CI values for different combinations of mPTEN and siAR, with values below 1 indicating synergistic effects and values below 0.5 indicating strong synergy. (E) Comparison of *f*_additive_ and *f*_combination_ values. ns, no significant difference; *, *p* < 0.05; **, *p* < 0.01; and ***, *p* < 0.001 (*n* = 4).

### 3.3 Apoptosis Analysis for siAR and mPTEN LNPs

To further study the potential enhanced cytotoxicity from the coadministration of siAR and mPTEN LNPs, apoptosis induction was evaluated in C4-2 PCa cells. Representative bright field and Calcein-AM staining images showed increased cell death with concurrent delivery of mPTEN and siAR LNPs compared to single-agent treatments, as evidenced by the decreased number of viable cells and reduced fluorescence signal (Figure 3A). Quantitative analysis of the MFI of the Calcein-AM signal indicated a significantly reduced live cell signal in the combination group, confirming enhanced cytotoxicity (Figure 3B).

**Figure 3:**
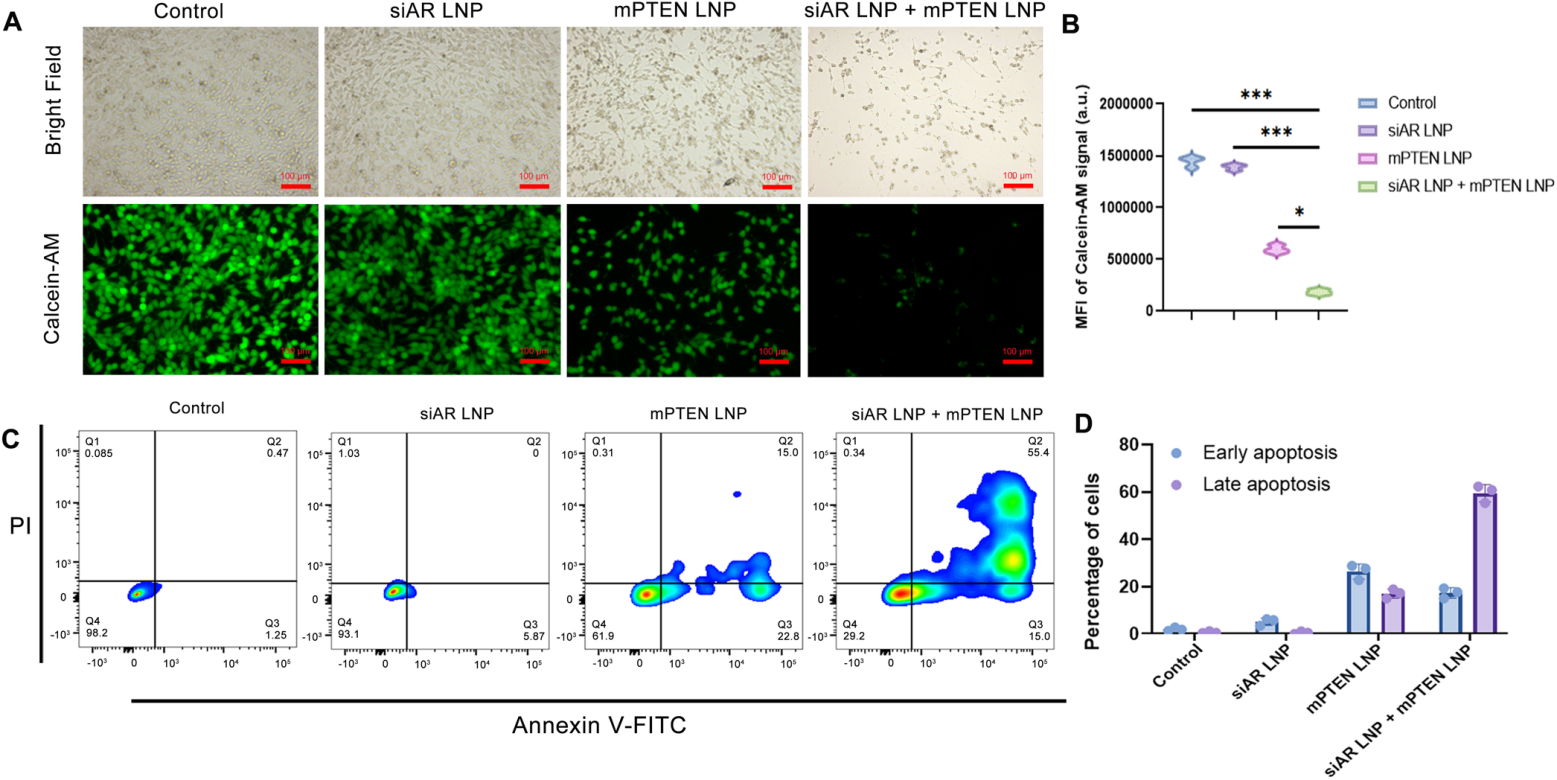
Apoptosis induction by concurrent delivery of siAR and mPTEN LNPs in C4-2 cells. (A) Representative bright field and Calcein-AM staining images of C4-2 cells after treatment with PBS (control), siAR LNP, mPTEN LNP, and coadministration of mPTEN and siAR (siAR LNP + mPTEN LNP). Scale bar: 100 μm. (B) MFI of Calcein-AM signal from (A). (C) Flow cytometry analysis using Annexin V-FITC and PI staining to assess apoptosis induced by the four groups. (D) Quantitative analysis of early and late apoptosis from flow cytometry results in (C). *, *p* < 0.05; **, *p* < 0.01; and ***, *p* < 0.001 (*n* = 4).

Flow cytometry analysis using Annexin V-FITC and PI staining was also performed to assess apoptosis. The results showed a significant increase in both early and late apoptotic cell populations in the combination treatment group compared with single-agent treatments (Figure 3C). Quantitative analysis of apoptosis revealed that the percentage of early and late apoptotic cells was significantly higher in the cotreatment group, with over 60% of cells undergoing apoptosis, compared to minimal or low apoptosis in the control and single-agent groups (Figure 3D). These results demonstrate that the enhanced cytotoxicity observed with the concurrent treatment of siAR and mPTEN LNPs is primarily due to the induction of much higher apoptosis. The combination treatment effectively triggers both early and late apoptosis, suggesting that the simultaneous inhibition of AR and restoration of PTEN may disrupt key survival pathways, leading to programmed cell death. These findings further support the synergistic effect of the dual-targeting strategy and highlight its potential as a promising therapeutic approach for the treatment of advanced PCa.

### 3.4 Mechanistic Study of the Synergistic Effect by siAR and mPTEN LNPs

To elucidate the mechanisms underlying the observed synergistic effect of the coadministration of siAR and mPTEN LNPs, Western blot analysis was performed to evaluate the expression of key proteins involved in PCa cell survival and apoptosis (Figure 4A). Co-treatment with siAR and mPTEN LNPs led to a marked decrease in AR, and the level of PTEN expression was successfully restored, as evidenced by increased PTEN protein levels. Phosphorylated AKT (pAKT) and phosphorylated ERK (pERK) levels were decreased, indicating effective suppression of the PI3K/AKT signaling pathway and inhibition of the ERK signaling pathway, which are known to promote cell survival and proliferation in PCa. The analysis also revealed increased levels of cleaved caspase-3 (c-Cas3), a marker of apoptosis, in the cotreatment group compared to single-agent treatments, indicating enhanced apoptosis induction.

**Figure 4:**
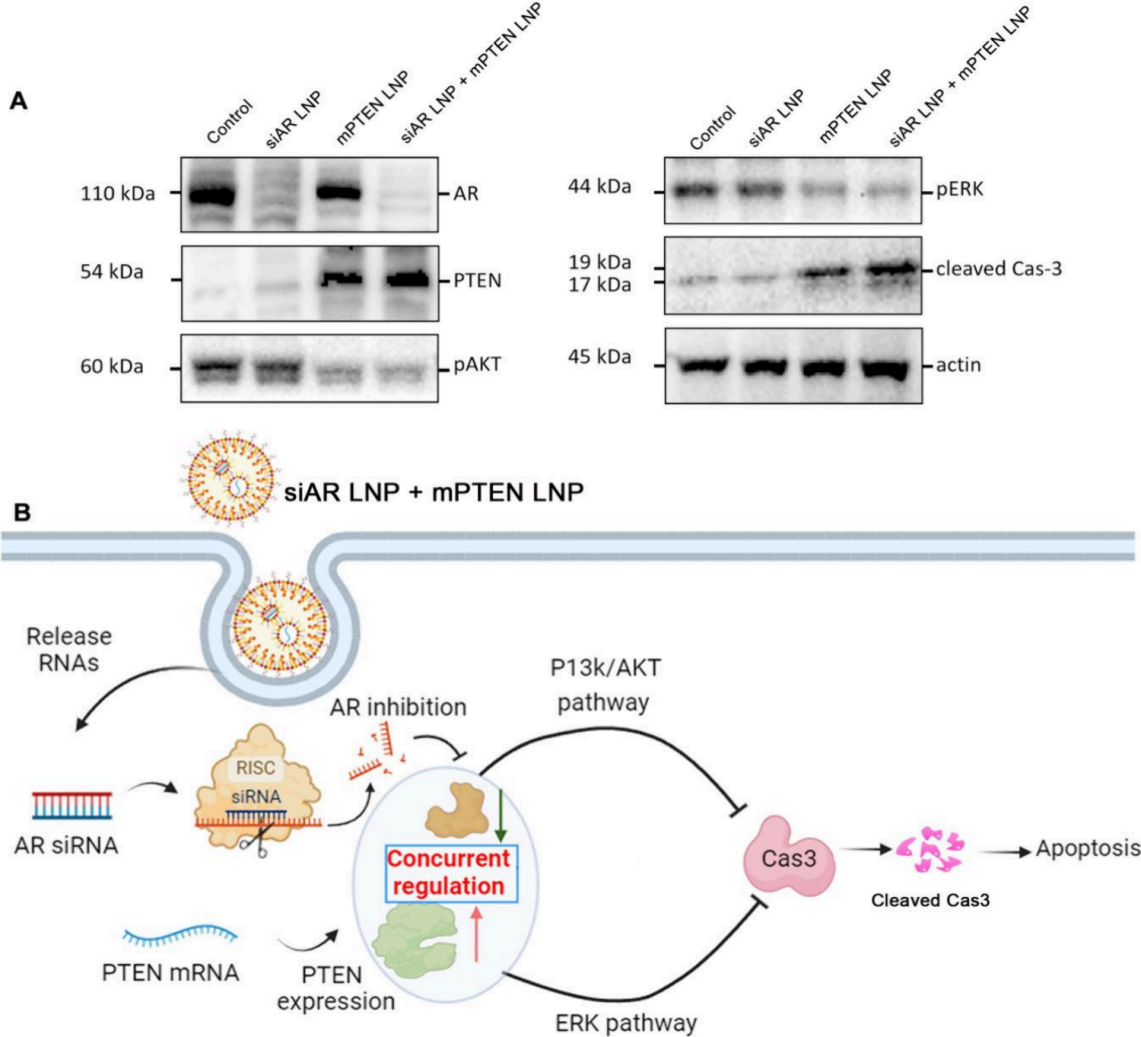
Mechanistic evaluation of siAR and mPTEN cotreatment in PCa cells. (A) Western blot analysis of AR, PTEN, pAKT, pERK, and c-Cas3 levels in C4-2 cells treated with siAR LNP, mPTEN LNP, or siAR LNP + mPTEN LNP. (B) Schematic representation of the proposed mechanism of action for coadministration of siAR and mPTEN LNPs.

The proposed mechanism of action for the coadministration of siAR and mPTEN LNPs is shown in Figure 4B. AR silencing leads to decreased AR-mediated transcriptional activity, while restoration of PTEN negatively regulates the PI3K/AKT and ERK pathways. By concurrently restoring PTEN to inhibit the PI3K/AKT pathway and silencing AR to inhibit the ERK pathway, we effectively block two major survival routes, thereby preventing compensatory signaling and enhancing proapoptotic responses through caspase-3 activation. We define concurrent regulation as the simultaneous modulation of two distinct cellular pathways that occur through different molecular mechanisms, each operating on its own time scale. In our system, mPTEN and siAR are coadministrated to achieve concurrent effects: restoration of PTEN tumor suppressor function and inhibition of the AR tumorigenic pathway in PCa cells. This dual-targeting approach effectively addresses both oncogenic drivers and tumor suppressor deficiencies, providing a comprehensive therapeutic effect against PCa.

### 3.5 LNP Codelivery of siAR and mPTEN

We also compared the efficacy of codelivery of siAR and mPTEN in the same LNP to coadministration of siAR LNP and mPTEN LNP, using siRNA and mRNA concentrations of 20 nM and 250 ng/mL, respectively. First, we performed EE measurement experiments with model siLuc and mGFP to confirm that each LNP contained the correct amount of siRNA and mRNA. We found that the EE of siLuc and mGFP was 70.27 and 92.23%, respectively, which is similar to the EE for each RNA in separate LNPs, showing effective RNA complexation. The results are presented in Figure S4, showing the cell viability in both C4-2 and LNCaP. The cell death for the codelivery strategy was 68.06% ± 5.32% and 70.90% ± 9.96% for C4-2 and LNCaP, respectively. On the other hand, the cell death for the combination treatment was 76.88% ± 11.62% and 70.11% ± 9.35% for C4-2 and LNCaP cells, respectively. The therapeutic efficacy of both approaches is comparable. These data suggest that LNP codelivery is a viable alternative strategy for siAR and mPTEN cotreatment. Given that codelivery in one particle may be beneficial for consistent pharmacokinetics and biodistribution of multiple payloads and for maintaining their synergistic ratio,^35^ the LNP codelivery strategy may be adopted for future in vivo studies to test the antitumor efficacy of concurrent AR silencing and PTEN restoration and their synergistic effect.

## 4 Conclusions

This study presents a novel approach to cancer therapy by concurrently delivering siRNA and mRNA to silence pro-tumorigenic drivers and restore tumor suppressor function. While concurrent siRNA and mRNA delivery has been shown as proof-of-concept,^36^ this study, to the best of our knowledge, is the first report of concurrent siRNA and mRNA therapy for cancer. Our results demonstrate that simultaneous silencing of AR and restoration of PTEN exhibit a strong synergistic effect in PCa cells. This strategy may also be robustly extended to other tumorigenic drivers (e.g., MYC) and tumor suppressors (e.g., p53) in PCa and other cancer types, such as breast cancer, non-small cell lung cancer, and hepatocellular carcinoma. With careful selection of pro-tumorigenic and tumor-suppressing pathways, the versatility of this codelivery system suggests broad applicability to various cancers, potentially improving outcomes through precise modulation of oncogenic and tumor-suppressive pathways.

In our experiments, we tested both the combined delivery of two LNPs and codelivery with one LNP. While combined delivery may potentially enable titration of the ratios of siRNA and mRNA in a more reproducible and predictable manner, codelivery could have the advantage of overlapping pharmacokinetics and biodistribution of both siRNA and mRNA. Additionally, while concurrent siRNA and mRNA therapy could be highly effective in vitro as demonstrated in this work, in vivo experiments (e.g., pharmacokinetics, biodistribution, efficacy, and safety) are still needed to further solidify it as a new viable strategy for cancer treatment. Additionally, an in-depth understanding of the synergy of siAR and mPTEN is still needed. To further clarify the mechanism behind this synergistic effect, comprehensive transcriptomic and proteomic analyses will be needed, which may lead to the discovery of new therapeutic targets.

In conclusion, our study provides strong evidence that concurrent delivery of siRNA to silence pro-tumorigenic drivers and mRNA to restore tumor suppressors could achieve synergistic antitumor effects. This RNA-based dual-targeting strategy holds significant potential for developing more effective treatments for advanced PCa and possibly other cancers. Future in vivo studies and a mechanistic understanding may further validate and enhance this unique therapeutic approach for its potential clinical development.

## Supplementary Material



## Notes

Size distribution of LNP formulations; cytotoxicity of siLuc LNP, mGFP LNP, and siLuc LNP + mGFP LNP; concurrent treatment of siAR and mPTEN LNPs in LNCaP cells; LNP codelivery of siAR and mPTEN in C4-2 and LNCaP cells; original Western blot images; and tables characterizing siRNA LNP and mRNA LNP formulations (PDF)
