# Supplementary material for: Lipid Nanoparticle Delivery of mRNA and siRNA for Concurrent Restoration of Tumor Suppressor and Inhibition of Tumorigenic Driver in Prostate Cancer
Source: ACS Nanosci Au. 2024 Dec 26;5(4):284–92. doi: 10.1021/acsnanoscienceau.4c00066 (PMC12371500; doi:10.1021/acsnanoscienceau.4c00066)
Supplement: Supplementary file 1 [file ng4c00066_si_001.pdf]

Lipid Nanoparticle Delivery of mRNA and siRNA for Concurrent Restoration of Tumor Suppressor and Inhibition of Tumorigenic Driver in Prostate Cancer

Ryan A Farokhzad[1], Jing Luo[2], Li Jia[2], Yang Zhang[1,*], Jinjun Shi[1,*]

[1] Center for Nanomedicine and Department of Anesthesiology, Perioperative and Pain Medicine, Brigham and Women's Hospital, Harvard Medical School, Boston, MA 02115, USA
[2] Department of Urology, Brigham and Women's Hospital, Harvard Medical School, Boston, MA 02115, USA

Supplementary Figures

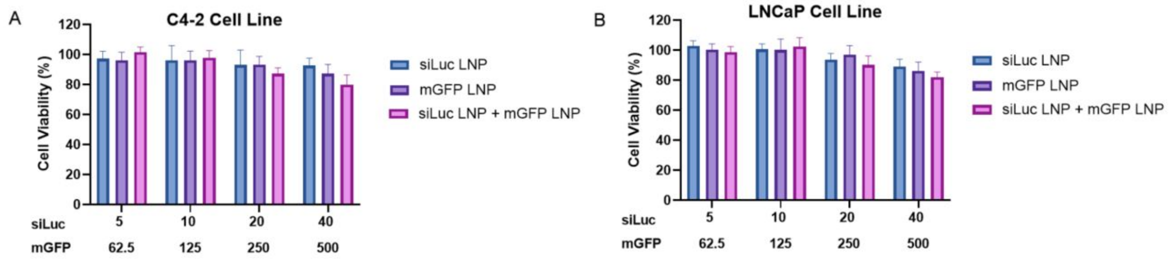

Figure S1. Cytotoxicity of siLuc LNPs, mGFP LNPs, and siLuc LNPs + mGFP LNPs with different RNA concentrations in (A) C4-2 and (B) LNCaP cells.

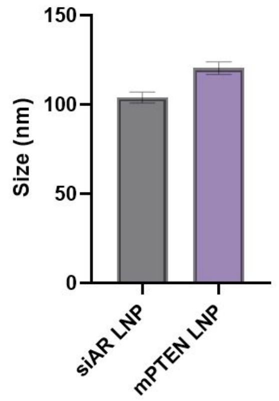

Figure S2. Average size of siAR LNPs and mPTEN LNPs.

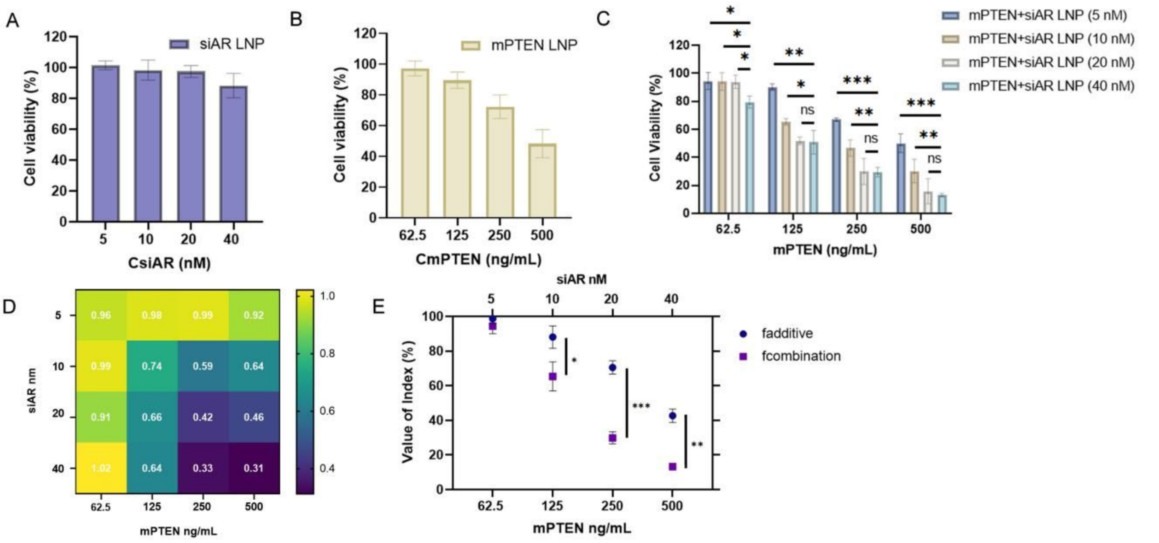

Figure S3. Concurrent treatment of siAR LNPs and mPTEN LNPs in LNCaP cells. (A, B) Dose-responsive cell viability of LNCaP cells after treatment with (A) siAR LNPs and (B) mPTEN LNPs at varying concentrations. (C) Cell viability following co-treatment with siAR LNPs and mPTEN LNPs at different siAR concentrations (5, 10, 20, and 40 nM) and mPTEN concentrations (62.5, 125, 250, and 500 ng/mL). (D) Heatmap showing CI values for different combinations of mPTEN and siAR, with values below 1 indicating synergistic effects and values below 0.5 indicating strong synergy. (E) Comparison of $f_{additive}$ and $f_{combination}$ values. ns, no significant difference; *, p<0.05; **, p<0.01; ***, p<0.001 (n = 4).

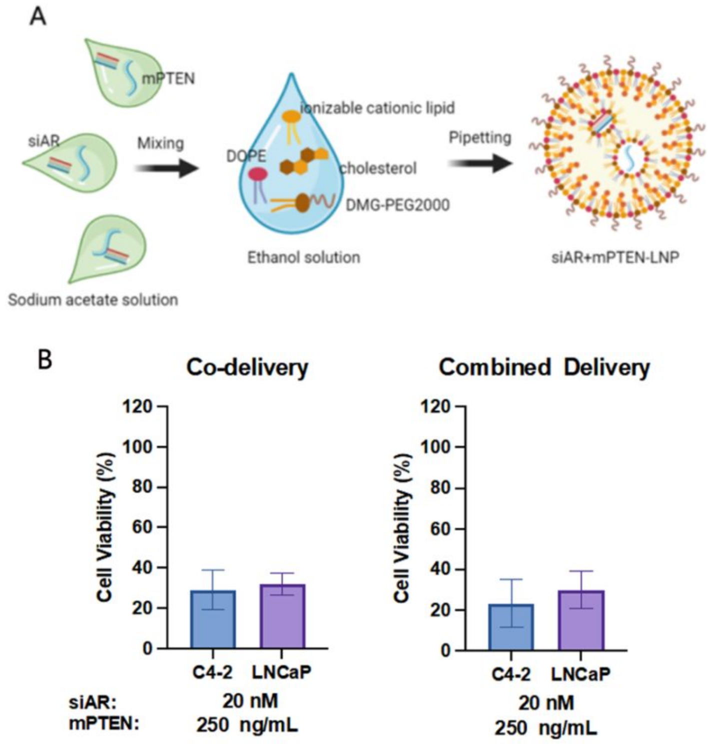

Figure S4. LNP co-delivery siAR and mPTEN. (A) Schematic of the formulation process for

siRNA+mPTEN LNPs. (B) Cell viability of co-delivery of siAR and mPTEN in one LNP vs. combined delivery of siAR LNP and mPTEN LNP in C4-2 and LNCaP cells.

Original WB images

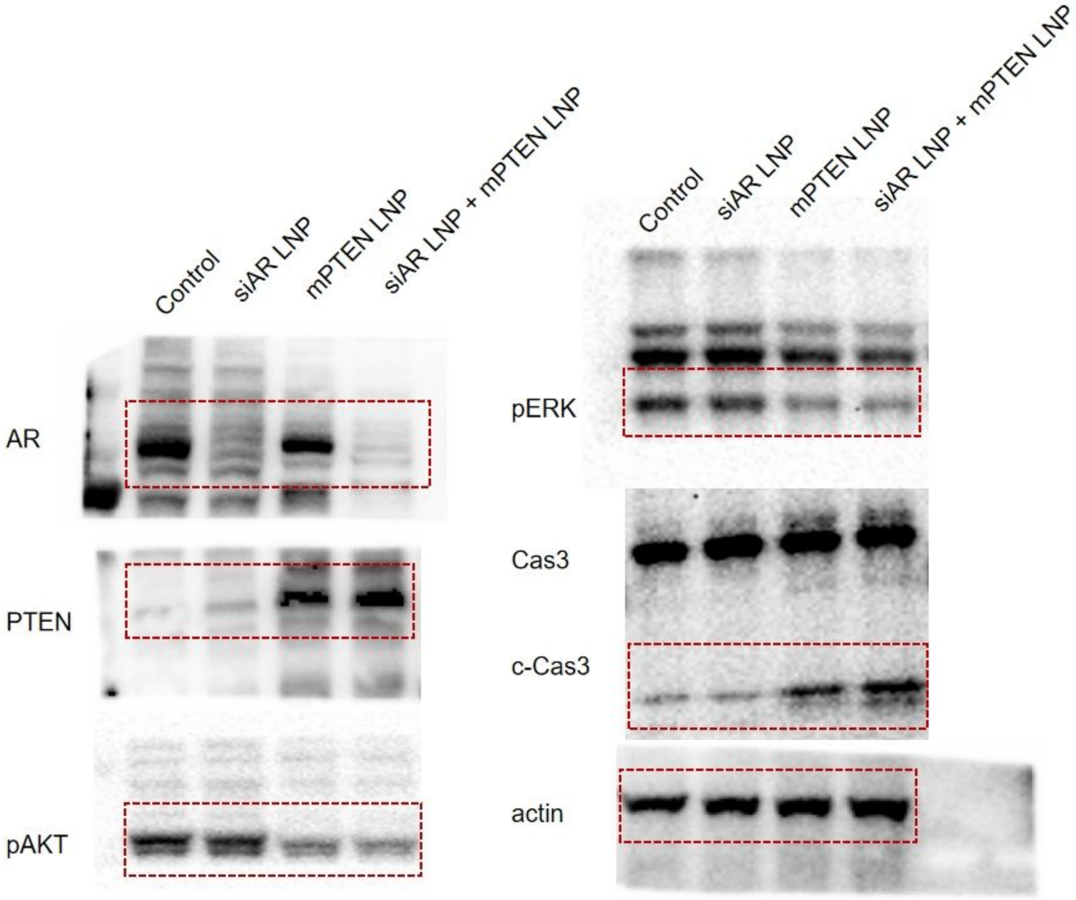

Table S1. siRNA LNP formulations with different ratios of DOPE, ionizable lipid, cholesterol, and DMG-PEG2000

Formulation	Factors (molar ratio)				Size (nm)	Zeta potential (mV)	EE (%)	Cell viability (%)
	**DOPE (%)**	**Ionizable lipids (%)**	**Chol (%)**	**DMG-PEG (%)**				
MC3-1	10	50	38.5	1.5	129.5	-2.57	70.55	93.5
MC3-2	15	35	48.5	1.5	111.1	-1.73	61.89	98.5
MC3-3	15	40	43.5	1.5	78.6	-4.75	69.30	92.6
MC3-4	13.5	50	35	1.5	106.4	-1.09	73.19	94.3
MC3-5	15	50	33.5	1.5	91.5	-4.14	66.78	90.3
MC3-6	18.5	50	30	1.5	131.7	-0.72	67.49	91.9
SM-102-1	10	50	38.5	1.5	124.1	0.44	69.09	101.6
SM-102-2	15	35	48.5	1.5	151.3	-3.23	58.55	105.8
SM-102-3	15	40	43.5	1.5	86.8	-1.74	74.69	98.6
SM-102-4	13.5	50	35	1.5	116.9	-0.58	75.59	97.1
SM-102-5	15	50	33.5	1.5	132.5	-5.60	61.29	93.6
SM-102-6	18.5	50	30	1.5	137.8	-4.39	66.48	95.8

EE: encapsulation efficiency

Table S2. mRNA LNP formulations with different ratios of DOPE, ionizable lipid, cholesterol, and DMG-PEG2000

Formulation	Factors (molar ratio)				Size (nm)	Zeta potential (mV)	EE (%)	Cell viability (%)
	**DOPE (%)**	**Ionizable lipids (%)**	**Chol (%)**	**DMG-PEG (%)**				
MC3-1	10	50	38.5	1.5	134.8	-8.98	90.79	92.8
MC3-2	15	35	48.5	1.5	111.2	-4.7	86.37	92.7
MC3-3	15	40	43.5	1.5	92.2	-3.09	90.72	93.8
MC3-4	13.5	50	35	1.5	108.7	-0.70	93.81	97.5
MC3-5	15	50	33.5	1.5	96.8	-2.75	89.94	93.6
MC3-6	18.5	50	30	1.5	134.6	-12.39	87.86	92.1
SM-102-1	10	50	38.5	1.5	131.4	-9.31	89.50	95.8
SM-102-2	15	35	48.5	1.5	143.6	-8.35	86.52	94.1
SM-102-3	15	40	43.5	1.5	88.5	-2.75	90.37	95.9
SM-102-4	13.5	50	35	1.5	127.4	-6.11	89.29	93.3
SM-102-5	15	50	33.5	1.5	132.5	-3.71	93.87	95.8
SM-102-6	18.5	50	30	1.5	130.6	-2.62	89.88	94.0

EE: encapsulation efficiency
